# Successful Scientific Writing and Publishing: A Step-by-Step Approach

**DOI:** 10.5888/pcd15.180085

**Published:** 2018-06-14

**Authors:** John K. Iskander, Sara Beth Wolicki, Rebecca T. Leeb, Paul Z. Siegel

**Affiliations:** 1 Centers for Disease Control and Prevention, Atlanta, Georgia; 2 Association of Schools and Programs of Public Health, Washington, District of Columbia

## Abstract

Scientific writing and publication are essential to advancing knowledge and practice in public health, but prospective authors face substantial challenges. Authors can overcome barriers, such as lack of understanding about scientific writing and the publishing process, with training and resources. The objective of this article is to provide guidance and practical recommendations to help both inexperienced and experienced authors working in public health settings to more efficiently publish the results of their work in the peer-reviewed literature. We include an overview of basic scientific writing principles, a detailed description of the sections of an original research article, and practical recommendations for selecting a journal and responding to peer review comments. The overall approach and strategies presented are intended to contribute to individual career development while also increasing the external validity of published literature and promoting quality public health science.

## Introduction

Publishing in the peer-reviewed literature is essential to advancing science and its translation to practice in public health (1,2). The public health workforce is diverse and practices in a variety of settings (3). For some public health professionals, writing and publishing the results of their work is a requirement. Others, such as program managers, policy makers, or health educators, may see publishing as being outside the scope of their responsibilities (4).

Disseminating new knowledge via writing and publishing is vital both to authors and to the field of public health (5). On an individual level, publishing is associated with professional development and career advancement (6). Publications share new research, results, and methods in a trusted format and advance scientific knowledge and practice (1,7). As more public health professionals are empowered to publish, the science and practice of public health will advance (1).

Unfortunately, prospective authors face barriers to publishing their work, including navigating the process of scientific writing and publishing, which can be time-consuming and cumbersome. Often, public health professionals lack both training opportunities and understanding of the process (8). To address these barriers and encourage public health professionals to publish their findings, the senior author (P.Z.S.) and others developed Successful Scientific Writing (SSW), a course about scientific writing and publishing. Over the past 30 years, this course has been taught to thousands of public health professionals, as well as hundreds of students at multiple graduate schools of public health. An unpublished longitudinal survey of course participants indicated that two-thirds agreed that SSW had helped them to publish a scientific manuscript or have a conference abstract accepted. The course content has been translated into this manuscript. The objective of this article is to provide prospective authors with the tools needed to write original research articles of high quality that have a good chance of being published.

## Basic Recommendations for Scientific Writing

Prospective authors need to know and tailor their writing to the audience. When writing for scientific journals, 4 fundamental recommendations are: clearly stating the usefulness of the study, formulating a key message, limiting unnecessary words, and using strategic sentence structure.

To demonstrate usefulness, focus on how the study addresses a meaningful gap in current knowledge or understanding. What critical piece of information does the study provide that will help solve an important public health problem? For example, if a particular group of people is at higher risk for a specific condition, but the magnitude of that risk is unknown, a study to quantify the risk could be important for measuring the population’s burden of disease.

Scientific articles should have a clear and concise take-home message. Typically, this is expressed in 1 to 2 sentences that summarize the main point of the paper. This message can be used to focus the presentation of background information, results, and discussion of findings. As an early step in the drafting of an article, we recommend writing out the take-home message and sharing it with co-authors for their review and comment. Authors who know their key point are better able to keep their writing within the scope of the article and present information more succinctly. Once an initial draft of the manuscript is complete, the take-home message can be used to review the content and remove needless words, sentences, or paragraphs.

Concise writing improves the clarity of an article. Including additional words or clauses can divert from the main message and confuse the reader. Additionally, journal articles are typically limited by word count. The most important words and phrases to eliminate are those that do not add meaning, or are duplicative. Often, cutting adjectives or parenthetical statements results in a more concise paper that is also easier to read.

Sentence structure strongly influences the readability and comprehension of journal articles. Twenty to 25 words is a reasonable range for maximum sentence length. Limit the number of clauses per sentence, and place the most important or relevant clause at the end of the sentence (9). Consider the sentences:

By using these tips and tricks, an author may write and publish an additional 2 articles a year.
An author may write and publish an additional 2 articles a year by using these tips and tricks.

The focus of the first sentence is on the impact of using the tips and tricks, that is, 2 more articles published per year. In contrast, the second sentence focuses on the tips and tricks themselves.

Authors should use the active voice whenever possible. Consider the following example:

Active voice: Authors who use the active voice write more clearly.
Passive voice: Clarity of writing is promoted by the use of the active voice.

The active voice specifies who is doing the action described in the sentence. Using the active voice improves clarity and understanding, and generally uses fewer words. Scientific writing includes both active and passive voice, but authors should be intentional with their use of either one.

## Sections of an Original Research Article

Original research articles make up most of the peer-reviewed literature (10), follow a standardized format, and are the focus of this article. The 4 main sections are the introduction, methods, results, and discussion, sometimes referred to by the initialism, IMRAD. These 4 sections are referred to as the *body* of an article. Two additional components of all peer-reviewed articles are the title and the abstract. Each section’s purpose and key components, along with specific recommendations for writing each section, are listed below.

**Title.** The purpose of a title is twofold: to provide an accurate and informative summary and to attract the target audience. Both prospective readers and database search engines use the title to screen articles for relevance (2). All titles should clearly state the topic being studied. The topic includes the who, what, when, and where of the study. Along with the topic, select 1 or 2 of the following items to include within the title: methods, results, conclusions, or named data set or study. The items chosen should emphasize what is new and useful about the study. Some sources recommend limiting the title to less than 150 characters (2). Articles with shorter titles are more frequently cited than articles with longer titles (11). Several title options are possible for the same study (Figure).

**Figure 1:**
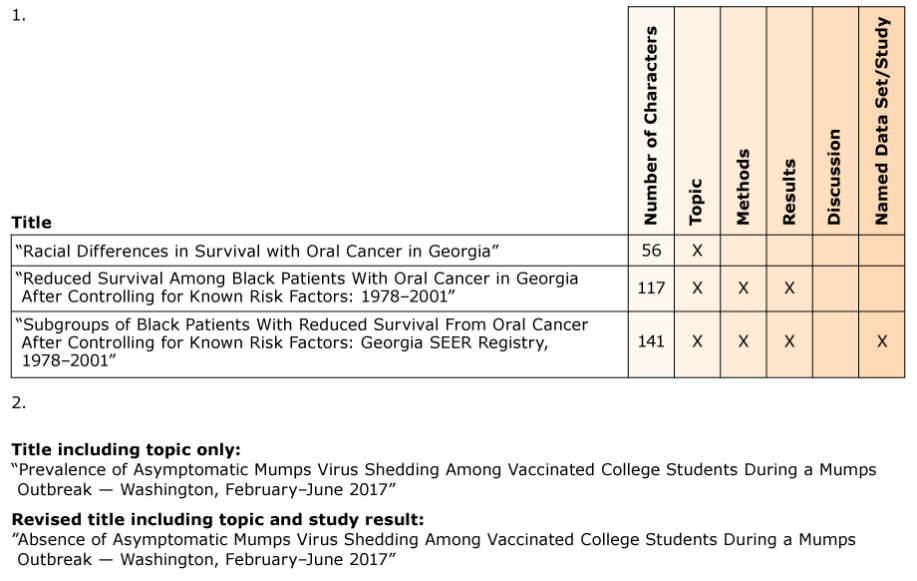
Two examples of title options for a single study.

**Abstract**. The abstract serves 2 key functions. Journals may screen articles for potential publication by using the abstract alone (12), and readers may use the abstract to decide whether to read further. Therefore, it is critical to produce an accurate and clear abstract that highlights the major purpose of the study, basic procedures, main findings, and principal conclusions (12). Most abstracts have a word limit and can be either structured following IMRAD, or unstructured. The abstract needs to stand alone from the article and tell the most important parts of the scientific story up front.

**Introduction**. The purpose of the introduction is to explain how the study sought to create knowledge that is new and useful. The introduction section may often require only 3 paragraphs. First, describe the scope, nature, or magnitude of the problem being addressed. Next, clearly articulate why better understanding this problem is useful, including what is currently known and the limitations of relevant previous studies. Finally, explain what the present study adds to the knowledge base. Explicitly state whether data were collected in a unique way or obtained from a previously unstudied data set or population. Presenting both the usefulness and novelty of the approach taken will prepare the reader for the remaining sections of the article.

**Methods**. The methods section provides the information necessary to allow others, given the same data, to recreate the analysis. It describes exactly how data relevant to the study purpose were collected, organized, and analyzed. The methods section describes the process of conducting the study — from how the sample was selected to which statistical methods were used to analyze the data. Authors should clearly name, define, and describe each study variable. Some journals allow detailed methods to be included in an appendix or supplementary document. If the analysis involves a commonly used public health data set, such as the Behavioral Risk Factor Surveillance System (13), general aspects of the data set can be provided to readers by using references. Because what was done is typically more important than who did it, use of the passive voice is often appropriate when describing methods. For example, “The study was a group randomized, controlled trial. A coin was tossed to select an intervention group and a control group.”

**Results**. The results section describes the main outcomes of the study or analysis but does not interpret the findings or place them in the context of previous research. It is important that the results be logically organized. Suggested organization strategies include presenting results pertaining to the entire population first, and then subgroup analyses, or presenting results according to increasing complexity of analysis, starting with demographic results before proceeding to univariate and multivariate analyses. Authors wishing to draw special attention to novel or unexpected results can present them first.

One strategy for writing the results section is to start by first drafting the figures and tables. Figures, which typically show trends or relationships, and tables, which show specific data points, should each support a main outcome of the study. Identify the figures and tables that best describe the findings and relate to the study’s purpose, and then develop 1 to 2 sentences summarizing each one. Data not relevant to the study purpose may be excluded, summarized briefly in the text, or included in supplemental data sets. When finalizing figures, ensure that axes are labeled and that readers can understand figures without having to refer to accompanying text.

**Discussion**. In the discussion section, authors interpret the results of their study within the context of both the related literature and the specific scientific gap the study was intended to fill. The discussion does not introduce results that were not presented in the results section. One way authors can focus their discussion is to limit this section to 4 paragraphs: start by reinforcing the study’s take-home message(s), contextualize key results within the relevant literature, state the study limitations, and lastly, make recommendations for further research or policy and practice changes. Authors can support assertions made in the discussion with either their own findings or by referencing related research. By interpreting their own study results and comparing them to others in the literature, authors can emphasize findings that are unique, useful, and relevant. Present study limitations clearly and without apology. Finally, state the implications of the study and provide recommendations or next steps, for example, further research into remaining gaps or changes to practice or policy. Statements or recommendations regarding policy may use the passive voice, especially in instances where the action to be taken is more important than who will implement the action.

## Beginning the Writing Process

The process of writing a scientific article occurs before, during, and after conducting the study or analyses. Conducting a literature review is crucial to confirm the existence of the evidence gap that the planned analysis seeks to fill. Because literature searches are often part of applying for research funding or developing a study protocol, the citations used in the grant application or study proposal can also be used in subsequent manuscripts. Full-text databases such as PubMed Central (14), NIH RePORT (15), and CDC Stacks (16) can be useful when performing literature reviews. Authors should familiarize themselves with databases that are accessible through their institution and any assistance that may be available from reference librarians or interlibrary loan systems. Using citation management software is one way to establish and maintain a working reference list. Authors should clearly understand the distinction between primary and secondary references, and ensure that they are knowledgeable about the content of any primary or secondary reference that they cite.

Review of the literature may continue while organizing the material and writing begins. One way to organize material is to create an outline for the paper. Another way is to begin drafting small sections of the article such as the introduction. Starting a preliminary draft forces authors to establish the scope of their analysis and clearly articulate what is new and novel about the study. Furthermore, using information from the study protocol or proposal allows authors to draft the methods and part of the results sections while the study is in progress. Planning potential data comparisons or drafting “table shells” will help to ensure that the study team has collected all the necessary data. Drafting these preliminary sections early during the writing process and seeking feedback from co-authors and colleagues may help authors avoid potential pitfalls, including misunderstandings about study objectives.

The next step is to conduct the study or analyses and use the resulting data to fill in the draft table shells. The initial results will most likely require secondary analyses, that is, exploring the data in ways in addition to those originally planned. Authors should ensure that they regularly update their methods section to describe all changes to data analysis.

After completing table shells, authors should summarize the key finding of each table or figure in a sentence or two. Presenting preliminary results at meetings, conferences, and internal seminars is an established way to solicit feedback. Authors should pay close attention to questions asked by the audience, treating them as an informal opportunity for peer review. On the basis of the questions and feedback received, authors can incorporate revisions and improvements into subsequent drafts of the manuscript.

The relevant literature should be revisited periodically while writing to ensure knowledge of the most recent publications about the manuscript topic. Authors should focus on content and key message during the process of writing the first draft and should not spend too much time on issues of grammar or style. Drafts, or portions of drafts, should be shared frequently with trusted colleagues. Their recommendations should be reviewed and incorporated when they will improve the manuscript’s overall clarity.

For most authors, revising drafts of the manuscript will be the most time-consuming task involved in writing a paper. By regularly checking in with coauthors and colleagues, authors can adopt a systematic approach to rewriting. When the author has completed a draft of the manuscript, he or she should revisit the key take-home message to ensure that it still matches the final data and analysis. At this point, final comments and approval of the manuscript by coauthors can be sought.

Authors should then seek to identify journals most likely to be interested in considering the study for publication. Initial questions to consider when selecting a journal include:

- Which audience is most interested in the paper’s message?
- Would clinicians, public health practitioners, policy makers, scientists, or a broader audience find this useful in their field or practice?
- Do colleagues have prior experience submitting a manuscript to this journal?
- Is the journal indexed and peer-reviewed?
- Is the journal subscription or open-access and are there any processing fees?
- How competitive is the journal?

Authors should seek to balance the desire to be published in a top-tier journal (eg, Journal of the American Medical Association, BMJ, or Lancet) against the statistical likelihood of rejection. Submitting the paper initially to a journal more focused on the paper’s target audience may result in a greater chance of acceptance, as well as more timely dissemination of findings that can be translated into practice. Most of the 50 to 75 manuscripts published each week by authors from the Centers for Disease Control and Prevention (CDC) are published in specialty and subspecialty journals, rather than in top-tier journals (17).

The target journal’s website will include author guidelines, which will contain specific information about format requirements (eg, font, line spacing, section order, reference style and limit, table and figure formatting), authorship criteria, article types, and word limits for articles and abstracts.

We recommend returning to the previously drafted abstract and ensuring that it complies with the journal’s format and word limit. Authors should also verify that any changes made to the methods or results sections during the article’s drafting are reflected in the final version of the abstract. The abstract should not be written hurriedly just before submitting the manuscript; it is often apparent to editors and reviewers when this has happened. A cover letter to accompany the submission should be drafted; new and useful findings and the key message should be included.

Before submitting the manuscript and cover letter, authors should perform a final check to ensure that their paper complies with all journal requirements. Journals may elect to reject certain submissions on the basis of review of the abstract, or may send them to peer reviewers (typically 2 or 3) for consultation. Occasionally, on the basis of peer reviews, the journal will request only minor changes before accepting the paper for publication. Much more frequently, authors will receive a request to revise and resubmit their manuscript, taking into account peer review comments. Authors should recognize that while revise-and-resubmit requests may state that the manuscript is not acceptable in its current form, this does not constitute a rejection of the article. Authors have several options in responding to peer review comments:

1. Performing additional analyses and updating the article appropriately
2. Declining to perform additional analyses, but providing an explanation (eg, because the requested analysis goes beyond the scope of the article)
3. Providing updated references
4. Acknowledging reviewer comments that are simply comments without making changes

In addition to submitting a revised manuscript, authors should include a cover letter in which they list peer reviewer comments, along with the revisions they have made to the manuscript and their reply to the comment. The tone of such letters should be thankful and polite, but authors should make clear areas of disagreement with peer reviewers, and explain why they disagree. During the peer review process, authors should continue to consult with colleagues, especially ones who have more experience with the specific journal or with the peer review process.

There is no secret to successful scientific writing and publishing. By adopting a systematic approach and by regularly seeking feedback from trusted colleagues throughout the study, writing, and article submission process, authors can increase their likelihood of not only publishing original research articles of high quality but also becoming more scientifically productive overall.
