# Erratum, Vol. 15, June 14 Release

**DOI:** 10.5888/pcd15.180085e

**Published:** 2018-10-18

The article “Successful Scientific Writing and Publishing: A Step-by-Step Approach,” published on June 14, 2018, contained inaccuracies in the acknowledgment of contributors. After consultation and mutual agreement among contributors and authors, corrections were made to the text and to Acknowledgments. The article was corrected on October 18, 2018, and can be found at https://www.cdc.gov/pcd/issues/2018/18_0085.htm. We regret any confusion or inconvenience these inaccuracies may have caused.

